# The use of online consultation tools for common mental health conditions in UK primary care: a qualitative interview study of patient and practitioner perspectives

**DOI:** 10.1186/s12875-026-03206-8

**Published:** 2026-02-05

**Authors:** Charlotte Archer, David Kessler, Louise Ting, Nicola Wiles, Katrina Turner

**Affiliations:** 1 Centre for Academic Mental Health, University of Bristol, Bristol Medical School, Bristol, UK; 2 Centre for Academic Primary Care, University of Bristol, Bristol Medical School, Bristol, UK; 3 National Institute for Health and Care Research, Applied Research Collaboration West, University Hospitals Bristol NHS Foundation Trust, Bristol, UK

**Keywords:** Digital health, Online consultations, Primary health care, Mental health, General practice, Qualitative research

## Abstract

**Background:**

UK general practices are now required to make online consultation tools available during practice hours. Evidence shows patients increasingly use them to access mental health support under the ‘digital first’ approach. Whilst they may increase time-efficiency for practices, we do not know whether practitioners and patients view them as a suitable consultation mode to discuss mental health. Our aim was to explore patients’ and practitioners’ views and experiences of using online consultation tools for mental health, to inform their future use.

**Method:**

In-depth interviews with 20 primary care practitioners and 21 patients. A topic guide was used to ensure consistency across interviews. Interviews were audio-recorded, transcribed verbatim, and analysed thematically. There was patient and public involvement throughout.

**Results:**

Patients and practitioners said online consultation tools encouraged reflective thinking about mental health and symptom disclosure. However, patients’ concerns around who might read the output meant they only provided limited information. Patients also reported online tools can be a barrier to accessing care, and those with less mental health literacy may struggle to articulate their concerns. Practitioners noted that continuity of care can be reduced when using online tools, and triage is more challenging if insufficient information is provided to determine if urgent care is needed.

**Conclusion:**

To ensure that online consultation tools do not increase inequity, they should remain part of a range of options for accessing mental health support in general practice and should not be a mandatory first step to access care. Online consultation tools can provide useful information for practitioners and may be more accessible than a telephone call for patients with anxiety or depression. However, practitioners may struggle to assess patient risk using these tools, which could mean patients do not receive the care they need. Patients might need support when first using online consultation tools and advice on who will access the information provided.

**Supplementary Information:**

The online version contains supplementary material available at 10.1186/s12875-026-03206-8.

## Introduction

From October 2025, all UK general practices are required to make online consultation tools available during practice hours [1]. Patients are often encouraged to use these tools when first contacting their practice to seek medical help, with some practices using a ‘digital first’ system whereby an online tool is the default approach to access care [2]. Patients can use these tools to complete a form to ask about symptoms or treatment, follow-up about a prior consultation, or make administrative requests such as referral letters. The information provided is read by practice staff, which may include receptionists, care navigators or clinical practitioners [3]. The aim is for patients to receive a response within one day, which may include booking an appointment, a prescription, or a request for further information [3]. Various tools are used in UK general practice, including eConsult (https://econsult.net/nhs-patients) and Accurx (https://www.accurx.com).

Online consultation forms are part of a range of digital measures introduced within the NHS to increase ways to access care, with over 30% of GP contacts taking place by telephone or online form in November 2023 [4]. The submission of these online forms has increased substantially, from 3.4 million in July 2024 to almost 6 million submissions in July 2025 [1]. Given that a large proportion (40% [5]) of primary care consultations involve the discussion of mental health [6], it is likely that a substantial proportion of online consultation forms also relate to this topic. Compared to consultations about physical health, patients seeking help for mental health conditions may face particular challenges including shame, stigma and discomfort with disclosure [7–9], alongside the importance of self-recognition and awareness of their own mental health [10]. Mental health conditions are also associated with issues of under detection, under diagnosis and complex risk assessment [11, 12].

Primary care review data suggest that online consultation tools may increase access for patients with mental health conditions, particularly those who may become anxious when speaking over the telephone [13]. Data also suggests they increase convenience for patients and potentially relieve pressure on GP services by automating appointment management, reducing phone calls and freeing up medical staff time [14]. However, there are concerns from practitioners that online tools impact the clinician-patient relationship by removing nonverbal communication, making rapport more difficult to establish [15], and interviews conducted with patients and practitioners suggest online consultations may restrict access to care by disadvantaging digitally-excluded patients or those with language barriers [16, 17]. Risk may also be underestimated if patients do not fully disclose the severity of their symptoms or if practitioners do not pick up on ‘red flags’ [14]. Importantly, online consultation tools may contribute to unmet healthcare needs by reducing continuity of care [18], which is a key component of effective primary mental health care [19].

Although online consultation tools can help practices triage care based on need, urgency and complexity, evidence suggests patients vary in their preference for using online systems to access care [20]. Also, little is known about access preferences for those who wish to discuss their mental health concerns with a primary care practitioner. Such concerns may be challenging to articulate in written form, and patients may have concerns around who may view the information they enter. Our aim was to understand practitioners’ and patients’ views and experiences of online tools for consultations about mental health, to inform their future use now they are a mandated service [1, 21].

## Method

This study was part of a larger project looking at the benefits and challenges of all modes of remote consultations for anxiety and depression, including telephone and video consultations [22]. This paper presents the data on online consultation tools for consulting about mental health, reported in line with the consolidated criteria for reporting of qualitative research (COREQ) (Supplement 1).

### Recruitment and sampling

Participants were recruited via GP practices located in Bristol and the surrounding area. Practices were informed about the project by the Clinical Research Network West of England (CRN WE). Details of practices who were interested in supporting the project were then passed onto the research team by the CRN. Practices were selected to support recruitment into the study based on maximising variation in the deprivation deciles of the practices, their rurality, the online consultation platforms they used and the sociodemographic characteristics of their patient populations (age, sex, ethnicity),. Practice managers or research leads informed practitioners in their practices about the research, with those interested in taking part contacting the research team directly. Initially only GPs and nurses were recruited. However, as data collection progressed it became apparent that other allied health professionals (AHPs) working in general practice also provided mental health care. Therefore, the study’s inclusion criteria was expanded to include AHPs, such as practice pharmacists and wellbeing coaches [23]. Practitioners provided details on their clinical role and their sociodemographic characteristics (age and gender) when contacting the research team. This information was then used to purposefully sample practitioners for interview.

GP practices searched their practice electronic databases to identify potential patients. Eligible patients were those aged ≥ 18 years, who had consulted at their practice (in any form) in the previous six months and had a common mental health condition (CMC), i.e. depression or anxiety. To exclude individuals who may have different needs or risks in relation to online consultation tools, practices excluded patients if they had bipolar disorder, schizophrenia, personality disorder, dementia, or substance (alcohol/drugs) misuse in the past year. Practices posted an invitation letter and an information sheet to patients they had identified as eligible to take part. Patients interested in being interviewed posted back a response form using a reply-paid envelope. The response forms asked for basic sociodemographic information (age, gender, and ethnicity), their CMC (diagnosis, duration, past episodes and medication), how they had accessed mental health care through their GP practice (in-person, telephone, videocall or online consultation tool) and their contact information. These details were used to purposively sample individuals of varying age, gender, ethnicity, CMC, modes of GP contact and who were registered with practices that differed in terms of deprivation decile. After variation was partially achieved on these characteristics, sampling then also considered clinical information such as the duration of the CMC and use of medication.

### Data collection

All the interviews were conducted by a researcher experienced in qualitative methods (CA). Interviews were held by telephone or videocall, and verbal consent to take part was audio-recorded prior to interview. CA introduced the project aims and told interviewees that she was not a clinician or an expert in online consultation tools. Two topic guides (one for practitioners and one for patients; supplement 2 & 3) were developed in parallel, and were used to ensure consistency across the interviews. They were based on the aims of the study and discussions within the research team and patient and public involvement (PPI) contributors. As part of the wider study, participants were asked about their views and experiences of a range of remote consultation mode for CMCs. Questions specific to online consultation tools asked about the benefits and challenges of use in initial and follow up appointments, when patients might or might not use them for mental health, and what could increase their accessibility or acceptability. After the interview, participants were asked to complete a brief demographic questionnaire (education, employment and marital status). This information was then used to describe those interviewed.

### Data analysis

Interviews were audio-recorded, transcribed verbatim, anonymised, and checked for accuracy. Data collection and analysis continued in parallel, with data collection ending when analysis of new data did not alter the themes we had constructed and data had generated sufficient insight and understanding to answer the research question, guided by the principles of information power [24] Analytical insights from early data collection informed later data collection. A reflective log was maintained throughout, with the researcher acknowledging her own disciplinary background in psychology and possible assumptions as someone who is confident in using online technology.

Reflexive thematic analysis was undertaken [25], to allow comparisons to be made within and across the patient and practitioner interviews. Each data set was analysed separately. Accounts from practitioners who were AHPs were also analysed separately from GPs, initially, and were then found to be similar in content. The first and last author (CA and KT) read and re-read a sample of transcripts from each dataset to identify possible codes. They then met to discuss their coding and interpretation of the data, and to develop two initial coding frameworks (one for each dataset). As new codes were identified in later transcripts, the coding frames were revised, and transcripts that had previously been coded were re-coded where necessary. Data analysis was supported by NVivo (version 12) software. An approach based on framework analysis [26] was then used to aid data interpretation. Data relating to specific codes were extracted and summarised in tables, which were then read and re-read to identify key themes and deviant cases. Once each data set had been fully analysed, the views and experiences of practitioners and patients were compared, allowing similarities and differences in views to be identified. As many themes were evident in both, in the results, patient and practitioners’ accounts are presented alongside each other. Where deviant cases were identified, they are reported as the views of ‘a few’ rather than ‘many’ or ‘most’.

### Patient and public involvement

All PPI contributors and the PPI co-investigator (LT) had lived experience of anxiety or depression and had experience of using online consultation tools for their mental health. Prior to the study starting, the PPI co-investigator, the study lead (CA) and one PPI contributor attended a meeting to discuss and comment on initial ideas for the study and to develop the patient-facing materials and interview topic guides. A third PPI contributor provided input via written comments. As a result of these comments, the wording used in the materials was revised so that they were easier to understand and the patient topic guide was designed to use a narrative format to enable each participant to share their lived experience in a chronological way. The PPI co-investigator was then involved throughout the research, attending meetings on recruitment, data analysis and interpretation of results. Seventeen months after the first meeting, the study lead, the PPI co-investigator and five PPI contributors met to discuss study findings, to further develop the themes and to identify the key messages for clinical practice. A sixth contributor shared written feedback. Their comments supported analysis and interpretation of the data. As a result of this input, a more balanced approach was taken in presentation of the themes, ensuring themes represented the positives and challenges of using online consultation tools.

## Results

Twenty practitioners from five different practices were interviewed between March 2023 and October 2023 (Table 1). Practitioner interviews lasted between 14 and 38 minutes. Just over half of the practitioners interviewed were female (*n* = 12, 60%), and the mean age was 43.2 years (standard deviation (SD) = 9.0). Those interviewed had been working in general practice between 1 and 36 years and 75% were GPs (*n* = 15). All practitioners regularly used online consultation tools.

**Table 1:** Characteristics of practitioners interviewed and practice deprivation deciles

Characteristic		*n*	%
*Gender*	*Female*	12	60
	*Male*	8	40
*Age*,* years*	*20–29*	1	5
	*30–39*	8	40
	*40–49*	7	35
	*50–59*	3	15
	*60+*	2	10
*Role in practice*	*Partner GP*	6	25
	*Salaried GP (including GP trainee)*	9	45
	*Allied health professional (AHP)*	5~	25
Practice deprivation score*	*1–3*	5	25
	*4–5*	2	10
	*6–7*	0	0
	*8–10*	13	65

~*n* = 3 pharmacists, *n* = 1 nurse and *n* = 1 wellbeing coach

*Deprivation score for the practice patient population where 1 indicates the most deprived patient population and 10 the least deprived. Taken from the National General Practice Profiles website [27] which calculated scores based on the 2015 English Indices of Deprivation [28].

Twenty-one patients from four different practices were interviewed between April 2023 and October 2023 (Table 2). The patient interviews lasted between 13 and 45 minutes. Just over half of those interviewed were female (*n* = 13, 65%), and the mean age was 49.7 years (SD = 16.9). Nearly all patients (*n* = 19) interviewed had experience of using online consultation tools, with just over half (*n* = 12) having used them for their mental health in the previous 12 months.

**Table 2:** Characteristics of patients interviewed and practice deprivation deciles

Characteristic		*n*	%
*Gender*	*Female*	13	61.9
	*Male*	8	38.1
*Age*,* years*	*18–29*	3	14.3
	*30–39*	4	19
	*40–49*	3	14.3
	*50–59*	3	14.3
	*60–69*	5	23.8
	*70+*	3	14.3
*Ethnicity*	*White*	15	71.4
	*Mixed*	3	14.3
	*Black*	1	4.8
	*Asian*	2	9.5
*Highest educational qualification*	*Higher diploma or degree or equivalent*	7	33.3
	*GCSE*,* O-level*,* A-level or equivalent*	11	52.4
	*No formal qualifications*	3	14.3
*Marital status*	Married/living as married	11	52.4
	Single	7	33.3
	Divorced	3	14.3
*Employment status*	*Paid employment*	12	57.1
	*Retired*	5	23.8
	*Unemployed due to ill health*	4	19
Practice deprivation score*	*1–3*	4	19
	*4–5*	4	19
	*6–7*	0	0
	*8–10*	13	61.9

*Deprivation score for the practice patient population where 1 indicates the most deprived patient population and 10 the least deprived. Taken from the National General Practice Profiles website [27] which calculated scores based on the 2015 English Indices of Deprivation [28]

**Fig. 1:**
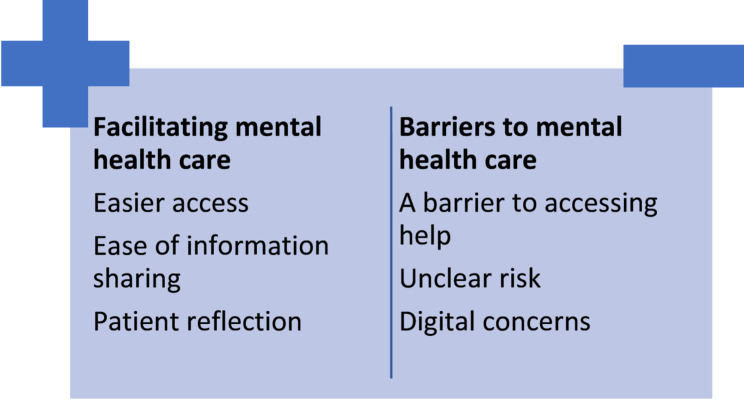
Barriers and facilitators for mental health care resulting from the use of online consultations tools in general practice

Findings from practitioner and patient interviews are presented together, under theme headings below and in Fig. 1. These themes reflect aspects of online consultation tools that were facilitators or barriers to mental health care. Age and deprivation were the only demographics that accounted for differences in perspectives, which are noted in two themes. We did not find differences relating to the other demographics. Quotes have been tagged with role, gender and age band.

### Facilitating easier access to mental health care

Practitioners and most patients were aligned in their views that online consultation tools could be more accessible for patients than having to call the practice, particularly for those who may not wish to discuss their mental health over the telephone. They were considered particularly helpful for perceived to be quick requests, like repeat medication requests, and administrative queries like extensions to fit notes.

*“Loads of them are at work basically so I think on the patients it makes us much more accessible…[they don’t have to] hide in the broom cupboard or something at work before they can talk about things.” GP 14*,* female*,* 40–49*.

*“Sometimes when motivation’s really hard and*,* you know*,* then thinking…I really do need to see my GP*,* I’ll make sure that’s a priority but I can’t call them until eight thirty tomorrow*,* that’s like a massive barrier*,* whereas if you could then and there…start that e-messaging consult thing*,* that’s really helpful”. Patient 10*,* female*,* 30–39*.

However, many practitioners explained that some patients submitted repeated consultations. They thought this was because online consultation tools were so accessible and reported the tendency to do this was particularly noticeable for patients who had severe anxiety or were struggling with their mental health.

*“The accessibility of it…patients who are really struggling with their mental health*,* they can end up sending in multiple requests.…people do have quite unstable mental health*,* they can bombard us with repeated messages about the same or related things which can be quite challenging.” AHP 3*,* male*,* 30–39*.

### Facilitating ease of information sharing

Many practitioners and patients shared the view that online consultation tools could be useful for sharing information about mental health, including informing risk assessment, history taking and patient preferences for treatment, prior to a patient consulting via telephone or in-person.

*“Before you speak to them you have already got this kind of round history of how they are feeling…what has made them sort of reach out…so you can just confirm it and things so it really helps get that background history.” AHP 5*,* female*,* 50–59*.

*“I put a request in to order more medication and then I left a note asking if I could increase it… and then I spoke to Dr W after*,* so it was really good…we got it going before I spoke to her”. Patient 21*,* male*,* 30–39*.

Many practitioners said that for anxious or nervous patients, prior use of online consultation tools could help the patient feel at ease during the subsequent consultation because they had already provided some information. Patients’ accounts also suggested they found it helpful to write their concerns in advance of a consultation. This was because doing so allowed the practitioner to initiate a discussion about how the patient was feeling, rather than the patient feeling under pressure to verbally explain this.

*“If you have got a fairly nervous patient…you start the consultation saying ‘thank you for sending an e-consult*,* I understand that you have got this…you have tried this…you are thinking about trying this’*,* because they have already told you.” GP 15*,* female*,* 30–39*.

*“Being able to go into a lot of detail online means that you skip a lot of the explaining stage when you have the appointment”. Patient 16*,* female*,* 18–29*.

### Easier to write concerns and promotes patient reflection

Some patients mentioned that they felt less pressure to explain their problem when writing compared with when they spoke to a receptionist over the telephone. They said that, particularly for sensitive or distressing issues, “*writing it is easier than saying it”* (Patient 7). Practitioners also noted this benefit.

*“[Patients] can write down things to tell us in advance and not everybody wants to talk about things but they’re happy to put it down*,* to type it down for you”. GP 14*,* female*,* 40–49*.

Some practitioners and patients had similar suggestions that completing an online consultations form could be therapeutic, promoting helpful reflection from the patient prior to the appointment.

“*[It’s] therapeutic…they have managed to be so detailed and spell out their feelings and then when you call them*,* it has given them almost homework to do and they have had a bit of thought and reflection on what they wrote*.*” GP 22*,* male*,* 20–29*.

*“What I find useful is you can put all the details down…without feeling like you are being rushed….the doctor can read it*,* so when they ring you back or whatever they have got all those notes…that is quite useful and obviously you can take your time to write it.” Patient 7*,* female*,* 40–49*.

### A barrier to accessing help

Practitioners working in a very deprived area said that the online consultation tool was not clearly displayed on their website, perhaps to reduce the number of inappropriate online consultation forms they were receiving. In contrast, some practices had a digital first system, whereby the patient had to use the online consultation tool before they could book an appointment or speak to a clinician. Some patients shared their frustration about having to complete this online form first. It felt like an additional step or barrier to accessing GP care.

*“I did do that [call the practice] and all they said was ‘you need to submit another Ask My GP’…it is just kind of like pushing you away…making it really difficult for you to get that mental help…Especially when you need it then and there.” Patient 14*,* female*,* 18–29*.

Some patients also described having to use online tools as feeling like they were being ‘fobbed off’, if they received a response from the practice rather than an appointment. Likewise, most practitioners were also aware that patients might hold these views, and suggested this could mean patients end up having repeat contacts. Both patients and practitioners noted that the conversation over online tools could feel very stilted, which had a subsequent impact on the mental health of the patient.

*“I often speak to people who’ve had that message [an online GP response to the information the patient has provided*,* signposting them to other services] and it hasn’t helped and all they felt is fobbed off and they come back a couple of months later a lot lower.” GP 13*,* male*,* 30–39*.

*“I was very emotional and very down in the dumps…I kept going on and emailing and emailing [to provide further information in response to practitioner responses]…it took them nearly over three weeks to see me…I just felt like I wasn’t getting any help or support.” Patient 14*,* female*,* 18–29*.

### Difficulties in triage due to unclear risk

Most practitioners described difficulties in being able to appropriately triage a patient when they had provided limited information on online consultation tools. They reflected that sometimes risk may be underestimated, meaning patients might not been seen as quickly as they needed to be. Some practitioners also noted that patients could sometimes exaggerate their symptoms, in order to be seen sooner. Similar to the experience shared by practitioners, some patients also described using certain words or phrases to be triaged more urgently.

*“The difficultly that we’ve got at the moment is whether they’re put into a routine or an urgent…and that’s where the problem lies if they haven’t put enough information on their initial Ask My GP for someone to triage it as urgent.” GP 11*,* male*,* 40–49*.

*“The thing that I’ve done quite a bit is use e-consult but…I have to kind of bend it (laughs) to make it work…it’s like quite a frustrating system…but if you kind of say like ‘I’m having a hard time’…that seems to get you a doctor’s appointment.” Patient 10*,* female*,* 30–39*.

Several practitioners noted that online consultation tools could reduce continuity of care, as any practitioner may triage the information provided, which could then make risk assessment more difficult if the practitioner did not know the patient. Likewise, practitioners working in practices located in more deprived areas noted the information provided by patients in online consultation tools might not be that useful if patients had poor health literacy or English was not their primary language. In keeping with this view, a few patients, from the same practices, indicated that they struggled to articulate their mental health concerns in words.

*“We are in the most deprived ward…there is some illiteracy and that cohort*,* they are probably just missing out because they do not know how to do it [complete the online form] properly…they don’t know how to advocate for themselves*,* if that makes sense. So you’ve potentially got some vulnerable groups where English may not be their first language*,* they may not know how to [provide sufficient information].” AHP 5*,* female*,* 50–59*.

*“They do e-consultations which I must admit I don’t like because…trying to express yourself*,* you know*,* in words…can be difficult.” Patient 12*,* female*,* 60–69*.

### Needing digital literacy and having privacy concerns

Nearly all patients who were older, who were not familiar with technology, said they were less comfortable completing online consultation forms. Patients explained that they were worried about how to use the tools, and found completing the form ‘stressful’, not knowing if it had been submitted or when they might get a response, which could add to their anxiousness. Correspondingly, most practitioners were also aware that older patients could have these concerns or a lack of understanding of the process.

*“I did all that [completing an online triage tool] because I wanted a GP appointment…it said you can wait up to four days…so I waited four days and I realised I obviously had not put the thing through at the end and pressed the button…technology is great but you have to follow the whole system through…it did not go through and you have lost it.” Patient 19*,* female 70–79*.

*“I would say probably our older patients like it less because they’re not used to it and the technology*,* I think can be more challenging.” GP 14*,* female 40–49*.

Some patients were also worried about who might read the information they submitted online and were not sure if it would be read by a receptionist, nurse or GP. They suggested online consultation tools lacked a ‘personal touch’ and wondered if the replies they received were artificial intelligence (AI) responses. For these reasons, they said they would rather speak to a healthcare professional over the telephone.

## Discussion

### Summary

Many practitioners and patients view online consultation tools as an accessible way to access mental health care, and which encourage reflective thinking, disclosure of symptoms and facilitates subsequent appointments. They also view online tools as enabling information around medical history or patient preferences for treatment to be gathered prior to a consultation. However, some patients think that online consultation tools are a barrier to accessing care and share concerns around who may read the information provided. Practitioners and patients explain that those with limited knowledge of technology, or are less able to express their feelings in writing, may struggle with accessing and using online forms. In addition, practitioners note that the link for the online consultation tools may not be clearly displayed on some practice websites. Practitioners suggest that continuity of care may be reduced when using this mode of consultation, and that there is often insufficient information to determine if the patient needs urgent mental health support, leading to challenges in triage.

### Strengths and limitations of the study

PPI informed all stages of the study and interviewing both practitioners and patients gave a comprehensive understanding of the implications of using online consultation tools in general practice to manage mental health conditions. Participants had a range of sociodemographic characteristics and backgrounds, although a smaller proportion were recruited from practices in more deprived areas. That said, sampling was sufficient to enable differences between the accounts of participants from different areas to be identified during analysis.

However, as patients had responded to invitations that stated the research was focused on alternative platforms for consultations in general practice, they may have held stronger views on such modes than their peers, particularly as most had experience of using online consultation tools. Indeed, less than a quarter of the sample were patients who described themselves as digitally disadvantaged by the use of online consultation tools. As all the interviews were conducted by telephone or by videocall, this may have been a barrier to participation for those who find remote engagement more difficult. Finally, given that all participants were located in Bristol and the surrounding areas, transferability of findings may be limited beyond the South West.

### Comparison with existing literature

The Department of Health have mandated that online consultation tools should be available during practice hours [1] with the General Medical Council contract stating that patients should be able to access care online, over the telephone or in-person [29]. Although there is no standardised model for how practices should use online consultation tools [29], our findings suggest some practices may be encouraging patients to access care solely through online tools (the ‘digital first’ approach) despite this not always being the preferred approach for patients with poor mental health. This is similar to research conducted prior to the pandemic, which found that GPs think online consultations offer increased opportunities for more patients to access care, but that patients have concerns around who reads their information and could feel ‘fobbed off’ if they only had online contact [16, 30].

There were some important facilitators for mental health care in relation to online consultation tools identified by patients and practitioners in our study. These included reflective thinking, patients feeling more at ease, and providing information prior to an appointment. The latter theme is in line with the international literature around online tools, in terms of them being useful for pre-appointment information gathering [31]. It is important to note that there is increasing use of AI in some practices to help the triage process [32]. This could be problematic considering practitioners in our study noted that mental health triage could be challenging where insufficient information had been provided by the patient using the tool. This reiterates the potential pitfalls of online consultation tools identified by others whereby, if practitioners are not able to determine severity of symptoms, practitioners are unable to accurately assess risk and provide appropriate safety netting advice [14, 15]. Alongside these studies, findings from the present study support evidence briefing recommendations that online systems should be co-designed with patients and that implementing AI-based systems should be avoided until their impact on inequalities and assessing risk is known [33].

Although the NHS suggests that online consultation tools can be beneficial for both patients and practices when they are adapted to meet local needs [34], findings from the present study suggest that further adaptions or education around using online consultation tools tailored to those with mental health conditions and local practice populations are needed. Views on the usefulness of online tools was largely dependent on the mental health literacy and technical knowledge of the patients in the present study, and this supports evidence from other studies that online tools may risk worsening existing inequalities, as they are not accessible by all patients [17, 35]. Patients seeking mental health support may have additional barriers such as concerns around stigma or feeling shame, which may impact help-seeking behaviour [8, 36], and concerns around acceptability of treatment [37]. They may also not recognise that their symptoms are related to their mental health [10]. Therefore, it is imperative that online consultation tools to do not further compound these barriers.

### Implications for research and/or practice

Online consultation tools can also provide useful information for practitioners and may be more accessible than a telephone call for patients with anxiety or depression. To ensure the use of online tools does not increase inequity and provides information that will enhance the consultation process, practices should not mandate the use of online consultation tools as a first step in accessing care, but rather continue to offer them as part of a range of options to contact the practice. Given that some patients may not value online consultation tools when seeking mental health support, or may use them inappropriately, practices may want to consider advising patients on how to best use online tools so they can receive suitable, timely and effective care. Practices should also indicate on their website and on the form itself, who will have access to the information provided and within what timeframe a patient will receive a response. The form could also include questions that will enable mental health risk to be better assessed. It is important for future research to replicate this study focusing on neurodivergent patients and with those with serious mental health conditions. Such individuals may have different risks and needs associated with using online consultation tools.

## Supplementary Information

Supplementary Material 1

Supplementary Material 2

## Data Availability

The datasets used and analysed during the current study are available from the corresponding author on reasonable request.

## Abbreviations

AHPs: Allied health professionals
AI: artificial intelligence
CMC: Common mental health condition
CRN WE: Clinical Research Network West of England
PPI: patient and public involvement
